# The multi-parameter optimized belief rule base for predicting student performance with interpretability

**DOI:** 10.1038/s41598-026-35950-3

**Published:** 2026-01-19

**Authors:** Jiaxing Li, Wenkai Zhou, Shilei Jiang, Tianhao Zhang, Xiping Duan, Ning Ma, Yuhe Wang

**Affiliations:** School of Computer Science and Information Engineering, Harbin Normal University, No.1 Shida Road, Limin Economic Development Zone, Harbin, Heilongjiang 150025 China

**Keywords:** Student performance prediction, Belief rule base, Interpretability, Evidential reasoning, Random forest, Engineering, Mathematics and computing

## Abstract

Predicting student performance is essential for making informed teaching decisions, customizing learning, and ensuring educational equity. When developing student performance prediction models, it is crucial to provide high prediction accuracy, a clear and logical prediction process, as well as easily understandable and traceable prediction outputs. The Belief Rule Base (BRB) combines expert knowledge to ensure accuracy while also having a certain degree of interpretability. However, the following problems still exist: When there are too many attributes, BRB will encounter the problem of rule combination explosion; After the optimization stage of the BRB model is completed, its interpretability may decline. Furthermore, when experts have limited knowledge, the reference values they cite may weaken the prediction accuracy of the model. In response to the above problems, this paper presents an interpretable student performance prediction model based on a multi-parameter optimized belief rule base(IBRB-m). Firstly, an attribute selection method based on random forest was introduced to screen out the important features that affect students’ academic performance; Secondly, The criteria for interpretability in the model optimization process have been defined. Finally, a student performance prediction model is constructed and a model parameter optimization method with multi-parameter optimization and interpretable constraints is proposed. The effectiveness of this method was verified through a case study of the performance of students in a certain school.

## Introduction

Student performance prediction refers to the process of using appropriate methodologies to anticipate students’future learning status,academic performance, or associated development trends by assessing the various types of behaviors, features,and performance data they exhibit during the learning process^1–3^. The student performance prediction model can assist educators in better understanding students’ individual differences and then implementing individualized teaching programs and tutoring methods to enhance students’ academic performance and learning outcomes^4,5^. Interpretable models are especially important for forecasting student performance because they can demonstrate the reasoning and reasons behind their conclusions, allowing proper judgments to be made^6^. Expert knowledge, as a key source of interpretability, should be included into the modelling process^7^. However, experts may make errors that greatly influence the accuracy of predictive models^8^. As a result, studying and developing highly accurate and interpretable prediction models of student performance is an important challenge in education^9,10^ .

In current research, models for predicting student performance can be categorized into three main types: (1) Black-box models. Constructing models based on observations, capable of handling complex data relationships and without requiring deep knowledge of the internal structure of the model, facilitates rapid development and deployment^11,12^. Li et al.^13^ researched and designed a tri-branch convolutional neural network structure combining row-based, column-based, and depth-based convolutional operations equipped with an attention mechanism. The architecture efficiently captures the consistency, orderliness, and time-based distribution of student behaviors in an end-to-end fashion, thereby enabling more precise behavioral analysis. Asif et al.^14^ proposed a more effective and accurate model for predicting the academic performance of students in Chinese-foreign cooperative education based on graph convolutional network (GCN). The model measures the similarity of students in academic performance through Pearson’s correlation coefficient and constructs an undirected graph to connect similar student nodes together. Through the learning of graph convolutional network, the model is able to effectively capture the correlation between students, thus improving the accuracy of grade prediction. Alamri and Alharbi^15^ introduced a deep learning-based model incorporating an attention mechanism for predicting student grades. The model makes full use of the feature extraction capability of deep learning and the advantages of the attention mechanism, and is able to identify and analyze the correlations between multiple factors that affect students’ performance. Meanwhile, the attention mechanism highlights the role of key factors, which improves the accuracy and interpretability of the prediction and supports the optimization of teaching strategies. Mingyu et al.^16^ proposed an end-to-end deep learning model that is able to automatically extract features from multiple sources of heterogeneous behavior-related data of students to achieve accurate prediction of scholastic achievemen. The core novelty of the model is the combination of a Long Short-Term Memory Network (LSTM) and a 2D Convolutional Network (2D-CNN): the time-dependent features of various types of behavioral data are captured by the LSTM, and the correlational features between different behaviors are extracted using the 2D-CNN, which comprehensively portrays students’ behavioral patterns and provides higher accuracy and reliability for prediction. This approach expands the toolset available to machine learning practitioners. These black-box models excel at processing large volumes of data.However, their internal mechanisms remain opaque to external users. The internal algorithms undergo a series of complex processes to generate predictions or classification results, without requiring users to understand the specific operating principles of the model^17^. Therefore, they may be less applicable in the pursuit of model interpretability. (2) White-box models. allow users to comprehensively grasp the operational logic and decision-making framework of the model, which facilitates debugging, optimization and trust building. Zhou et al.^18^ used data mining methods to analyze undergraduate student performance, focusing on two main areas. First, a decision tree model was used to predict students’ academic performance at the conclusion of four years of study, based on students’ historical data and key characteristics. Second, typical student progress is studied and combined with academic performance prediction in order to gain insights into students’ learning paths and support personalized educational interventions. Yang and Xu^19^ investigated interpretable models on student performance prediction. By analyzing and synthesizing the main research results, these models were classified according to nine dimensions. The results of the analysis showed that further research is still needed for interpretable models for student achievement prediction, especially on how to quantify and assess their interpretability while ensuring accuracy to maintain a balance between reliability and transparency of the prediction models. The inner structure and algorithms of white-box models are visible and do not depend on observations. However, its internal structure is complex and difficult to adapt to changes in data distribution, and it is less adaptable to dynamically changing data environments, resulting in lower accuracy. (3) Grey-box models. The grey-box model is a system analysis and design method that lies between the white box model and the black box model. It improves the accuracy of the model by constructing a model framework based on the model principle and adjusting and improving it using data samples, both to ensure the accuracy of the model and simultaneously to keep the modeling process somewhat interpretable^20–22^.

The belief rule base(BRB) is a typical grey-box model that inherits the advantages of black-box and white-box models, is compatible with both data-driven and knowledge-driven approaches, and is able to achieve data-driven model improvement by learning and optimizing from historical data in the presence of insufficient expert knowledge^23–25^. This fusion allows the system to utilize a priori knowledge as well as adapt to actual data. Compared to black-box models, the belief rule base is highly interpretable while maintaining high accuracy. Its rules and belief distributions can visualize the inference process, which is easy to verify and adjust. Compared with the white box model, BRB has high accuracy, and the historical data can be used to train and optimize the belief distributions as well as the rule parameters, so that the model can adapt to the dynamic changes of the complex environment. By filling knowledge gaps with data, it can also operate efficiently in scenarios where expert knowledge is insufficient or the formula cannot be clarified^26–28^. However, the model also has certain problems. In the traditional BRB-based student performance prediction model, when the model contains an overly large number of attributes, it may cause the problem of combinatorial explosion of rules . Meanwhile, traditional belief rule base optimization algorithms include the Whale Optimization Algorithm (WOA), the Differential Evolution (DE) algorithm, and the Projection Covariance Matrix Adaptive Evolution Strategy (P-CMA-ES). The randomness of these algorithms can cause significant differences between the prediction results and expert knowledge. Highly interpretable models can clearly display the logic and reasons behind the predictions, enabling teachers, students and parents to trust the conclusions of the models and avoid the distrust brought about by “black box models”. Furthermore, traditional belief rule base optimization algorithms often only focus on the optimization of belief levels and do not consider the influence of other parameters on the prediction results.

To address these issues, this paper proposes an interpretable student performance prediction method based on the Multi-parameter Optimized Belief Rule Base (IBRB-m). The main contributions of this paper are as follows:

1. A feature selection approach is introduced to reduce the complexity of the model and avoid the combinatorial explosion of rules. This approach reduces the complexity of the model by constructing multiple decision trees to evaluate the contribution of individual features, thus effectively selecting the features that are most critical for prediction model.
2. Based on the traditional interpretability criteria, the interpretability optimization criteria of the model were defined according to the characteristics of students’ academic performance, providing guidance for the construction process of the IBRB-m model.
3. A multi-parameter optimization and constraint modeling method is proposed, which effectively improves model accuracy.

The structure of the rest of this article is as follows. Section 2 summarizes the issues that need to be considered in the IBRB-m model. Section 3 describes the main process of constructing an IBRB-m model. In Sect. 4, the validity of the model is verified through a case study of the performance of students in a school. Finally, summarize the entire text.

## Problem description

The three following points need to be considered for the IBRB-m based predictive modeling of student performance:

**Problem 1:** How to resolve the problem of rules combination explosion, which arises due to a large number of attributes^29^. To circumvent this issue, employing an effective feature selection technique to filter out key attributes becomes crucial. Feature selection is a key step to enhance the effectiveness of the model. Appropriate feature selection not only enhances the model’s ability to recognize a different categories, but also minimizes the negative impact of irrelevant features on model performance. The feature selection procedure is illustrated in Eq. (1).

1 $$\begin{aligned} \{ x_1^{\prime }, x_2^{\prime }, \ldots , x_l^{\prime } \} = \text {Select}(x_1, x_2, \ldots , x_q) \end{aligned}$$

where $\text {Select}(\cdot )$ is the process of selecting the important attributes, $x_1$ is the first attribute, $x_2$ is the second attribute, $x_{\textrm{q}}$ is the $\textrm{q}_{\textrm{th}}$ attribute, and $\left\{ x_1^{\prime }, x_2^{\prime }, \ldots , x_l^{\prime }\right\}$ is the top *l* attributes with the highest importance. In this process, the top *l* features are chosen as inputs to the model from the most important features related to the output.

**Problem 2:** How to ensure interpretability of student performance predictions. Models that lack interpretability are not trustworthy in their outputs, and users may be skeptical of the model’s results, thus reducing trust in educational decisions. Therefore, it is necessary to take an in-depth analysis of students’ daily behavior and, accordingly, establish a number of basic interpretability principles to be followed in the modeling process, which can be expressed as a collection of key elements (2).

2 $$\begin{aligned} \text{ principle } :\left\{ p \mid p_1, p_2, \ldots , p_c\right\} \end{aligned}$$

where *p* represents the interpretable definition criteria and *c* represents the number of criteria.

**Problem 3:**How to optimize models with limited expert knowledge and the introduction of interpretability criteria. Considering that expert knowledge is inherently uncertain and the problem of insufficient expert knowledge, it is necessary to adjust the initial parameters of the model. The existing optimization algorithms of belief rule bases often only focus on the optimization of belief levels, while ignoring the influence of other parameters on the prediction results. Furthermore, the randomness of the optimization algorithm often undermines the interpretability of the model, resulting in significant differences between the optimized model rules and the original rules based on expert experience. Therefore, it is particularly important to select appropriate optimization parameters and design an optimization process with interpretability constraints. The specific description is as shown in Eq.  (3).

3 $$\begin{aligned} \gamma _{\text{ best } }=\operatorname {optimize}(x, y, \gamma , p) \end{aligned}$$

where optimize $(\cdot )$ is the optimization function, *x* is a group of input attributes, *y* is the prediction result, $\gamma$ is the collection of model parameters to be optimized, $\gamma _{\text{ best } }$is the series of optimized parameters of the model, and *p* is the interpretability criterion to be followed during the optimization process.

## Construction of a predictive model of student performance based on IBRB-m

**Fig. 1:**
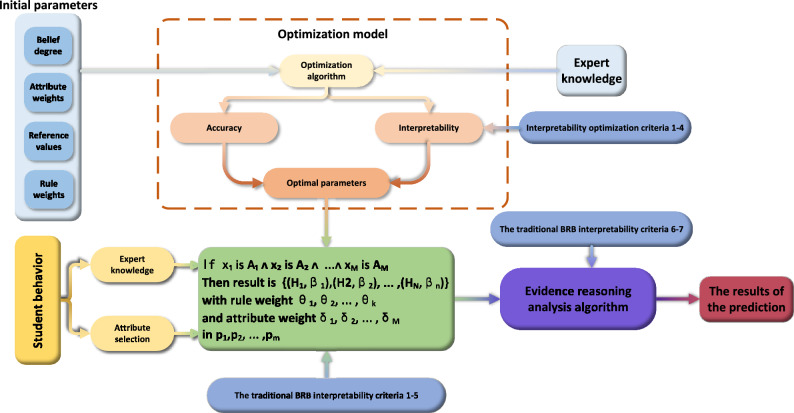
The student performance prediction model based on IBRB-m.

Accurate projection of student performance is crucial for informing educational decision-making and optimizing instructional strategies. Having an interpretable student performance prediction model can help educators and policy makers to understand the key factors affecting student performance so as to develop more targeted interventions. This paper proposes an IBRB-m based student performance predicting model to ensure that the predictive model retains accuracy and interpretability. In traditional BRB-based applications, excessive input attributes can cause rule combinatorial explosion problems. Considering the multi-attribute nature of student performance, an attribute selection module is added to identify the most critical features for model prediction and simplify the model. Next, the interpretability criteria relevant to student performance prediction are incorporated to construct and optimize the model. Meanwhile, integrate the reference values, belief levels, rule weights and attribute weights into the module to be optimized. The model structure is illustrated in Fig. 1. Section 3.1 outlines the feature selection methodology; Section 3.2 establishes interpretability standards for the IBRB-m based model; Section 3.3 describes the IBRB-m based framework for predicting student performance; Sections 3.4 and 3.5 detail the model’s inference and optimization procedures, respectively.

### Random forest based feature selection

In this article, the Random Forest algorithm is used to identify and select features that are important for predicting goals^30^. The random forest based feature selection process involves constructing an integrated model consisting of multiple randomized decision trees. In this process, every decision tree is trained using a randomly selected subset of features and samples to predict the target variables. During the construction process, each decision tree evaluates the effectiveness of each feature in the process of reducing data impurity. In this way, the importance of each feature is quantified, usually measured by its average contribution across all decision trees. These importance scores are then used to select the most important features that are most critical for improving prediction performance in random forests. The end result is a feature set consisting of the features that contribute the most to the prediction task, thus improving the overall performance of the model^31^. The steps are detailed as follows:

Step 1: For each decision tree in a random forest^32^, a subset of data is chosen from the original dataset through bootstrap sampling to serve as the training set. This process leaves about 1/3 of the data uninvolved in training, this is referred to as out-of-bag (OOB) data. The OOB data is utilized to assess the decision tree’s performance, and the resulting error is computed as the OOB data error, denoted $e r r_r^{O O B 1}$. This approach provides an unbiased estimation, and therefore usually does not require additional cross-validation or test sets to evaluate the model.

Step 2: Next, random noise interference is introduced to the sample features in the out-of-bag data, which means randomly altering the values of the sample features, and then reconstruct decision trees using the same random forest algorithm, after which we calculate the new out-of-bag data error, denoted as $e r r_{r, i}^{O O B 2}$.

Step 3: If the random forest consists of trees, the importance of features can be calculated through Eq. (4).

4 $$\begin{aligned} F I_{X_i}=\frac{1}{K} \sum _{r=1}^R\left( e r r_r^{O O B 1}-e r r_{r, i}^{O O B 2}\right) \end{aligned}$$

where *K* is the number of samples; $F I_{X_i}$ stands the importance of the $i_{t h}$ attribute.

The reason why this value reflects the importance of the feature is that if feature *X* is important for the prediction of the sample, the model will lose the information of this feature when random noise is added, resulting in a decrease in the prediction accuracy, That is, as $e r r_{r, i}^{O O B 2}$ increases. Therefore, the importance of *X* is proportional to the increase in $e r r_{r, i}^{O O B 2}$ compared to $e r r_r^{O O B 1}$.

Step 4: Calculate the out-of-bag error rate for each feature, and sort the importance of features in descending order according to the error rate. The features with low importance are eliminated by iteration until the optimal subset of features is selected.

**Algorithm Summary:** To clearly illustrate the feature selection process, Algorithm 1 presents the pseudocode for random forest-based feature selection.

**Figure:**
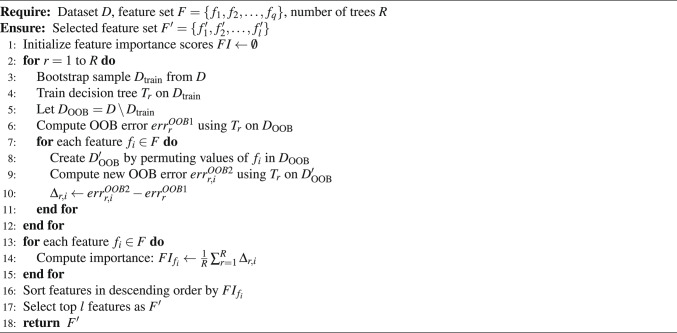
Algorithm 1 Random forest feature selection

### Interpretable criteria definitions of the prediction model

**Fig. 2:**
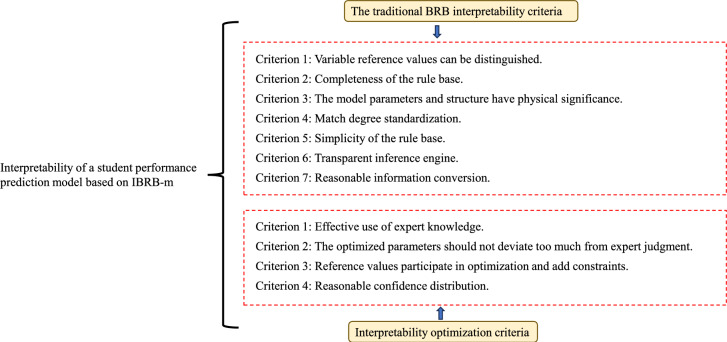
Interpretability criteria for the IBRB-m based model.

To ensure the model’s interpretability, it is essential to develop effective interpretability modeling criteria. Cao et al.^33^ defined general interpretability criteria, which can be seen in criteria 1–7. This paper presents several interpretability optimization criteria applicable to student performance prediction, as shown in optimization criteria 1–4. As shown in Fig. 2. The specific descriptions are as follows:

#### The traditional BRB interpretability criteria

Criterion 1: The reference values of the variables can be differentiated.

In models predicting student performance, reference values indicate varying levels or states of an attribute, making rule interpretations clearer. These values must encompass the entire spectrum of attribute values, with each representing a distinct level or state.

Criterion 2: Completeness of the rule base.

The completeness of the rule base requires the existence of at least one reference value corresponding to all possible input data, while ensuring that at least one rule is activated.

Criterion 3: The model parameters and structure are physically meaningful.

In an interpretable model, physical meaning is an important basis for its interpretability. If the parameters lack clear meaning, then the entire model loses its value. IBRB-m based models involve parameters such as belief degree, rule weights, attribute weights, and activation weights, each of which carries a specific physical or conceptual meaning.

Criterion 4: Match standardization.

Normalization of the degree of match helps to analyze the characteristics of a sample of student performance data, thus providing guidance for building an accurate performance prediction model. In a IBRB-m based model, the sum of the degrees of match between the current input data and the reference values should be limited to a range from 0 to 1.

Criterion 5: Simplicity of the rule base.

In components based on IBRB-m model, a concise rule base is the key to its interpretability. This helps researchers and developers to easily grasp the operation of the entire system and boost the performance of the model. Typically, a simple rule base contains fewer rules and fewer pre- and post-component parameters for each rule. However, the simplicity of a rule base can also limit IBRB-m applications. Therefore, building a reasonable structure or performing feature screening is a viable strategy to strengthen the performance of IBRB-m based models.

Criterion 6: Transparent inference engines.

Choosing a transparent inference machine is necessary. Belief rules are the basis of reasoning, and effectively applying these rules to generate predictions is the core issue. The ER algorithm synthesizes prediction results through precise mathematical operations combined with probabilities. Employing the ER algorithm as an inference mechanism to illustrate the reasoning process based on the IBRB-m model.

Criterion 7: Reasonable conversion of information.

In the inference phase, the system should ensure the integrity of the initial data as much as possible and perform reasonable information transformation within the belief structure. The ER method, grounded in rule and utility analysis, is an efficient algorithm as it enables equivalent and logical information transformation within the belief system.

####  Interpretability optimization criteria

Criterion 1: Effective utilization of expert knowledge.

Expert knowledge, derived from in-depth analysis and extensive practice, is vital for enhancing model interpretability. Model optimization often relies on a local search process based on initial expert assessments. Thus, integrating expert insights into model parameters and initial optimization solutions can effectively guide the process. This approach can more efficiently extract valuable information from a broader search space. The specific process is shown in the Eq. (5).

5 $$\begin{aligned} m^{(g)}=\left\{ \begin{array}{lc} E K & \text{ if } \textrm{g}=1 \\ m^{(g)} & \text{ if } \textrm{g} \ne 1 \end{array}\right. \end{aligned}$$

where $m^{(g)}$ is the first g band population.; *EK* stands for expert knowledge; Criterion 8 ensures initial interpretability by translating expert knowledge into parameters.

Criterion 2: Optimized parameters should not deviate excessively from expert judgment.

The parameters of the IBRB-m based model directly reflect the physical or statistical laws behind it. Unlike the difficult-to-understand black-box model, the IBRB-m based model is easier for users to understand and accept. By optimizing parameters and adding constraints, we can ensure that these key parameters are fully utilized in the model. The parameter constraints of the model are shown in Formula (6).

6 $$\begin{aligned} \begin{array}{rl} p_{l p} \le p \le p_{u p}:\left\{ \theta _{l p_k} \le \theta _k \le \theta _{u p_k}\right. \hspace{5.0pt}& \textrm{k} \in \{1,2, \ldots , \mathrm {~L}\}, \\ \delta _{l p_i} \le \delta _i \le \delta _{u p_i} \hspace{5.0pt}& \textrm{n} \in \{1,2, \ldots , \mathrm {~N}\}, \\ \beta _{l p_{k, n}} \le \beta _{k, n} \le \beta _{u p_{k, n}} \hspace{5.0pt}& \left. \textrm{i}, \mathrm {~m} \in \left\{ 1,2, \ldots , \mathrm {~T}_k\right\} \right\} \end{array} \end{aligned}$$

where $p_{l p}$ is the smallest value of the parameter. and $p_{u p}$ is the largest value of the parameter. $\theta _{l p_k}$ is the lower bound of the rule weight. $\theta _{u p_k}$is the upper bound of the rule weight. $\delta _{l p_i}$ is the lower bound of the attribute weight. $\delta _{u p_i}$ is the upper bound of the attribute weight. $\beta _{l p_{k, n}}$ is the lower bound of the belief distribution. $\beta _{u p_{k, n}}$ is the upper bound of the belief distribution. The parameters of the IBRB-m based model are reasonably constrained to prevent over-optimization leading to decline in model precision.

Criterion 3: Reference values participate in optimization and add constraints.

In the process of modeling and optimization, reference values, as part of expert knowledge, also have certain limitations. Reference values are crucial to improve the accuracy of model predictions. In view of this, this study proposes to integrate reference values into the optimization process. By introducing interpretability constraints, it can be ensured that the model obtains the optimal parameter settings based on a full reference to expert knowledge. This approach enhances the interpretability of the model while ensuring its accuracy. The constraints are shown in Formula (7).

7 $$\begin{aligned} V_{l p_u}<V_u<V_{u p_u} \quad \textrm{u} \in \{1,2, \ldots , \textrm{Z}\} \end{aligned}$$

where $V_{l p_u}$ is the minimum value of the reference value; $V_{u p_u}$ is the maximum value of the reference value; Z is the number of reference values to be optimized.

Criterion 4: Reasonable belief distributions.

**Fig. 3:**
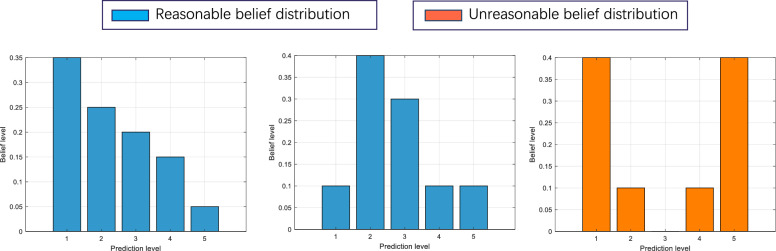
Comparison of reasonable and unreasonable belief distributions.

By integrating expert knowledge into the model as parameters through belief rules, the model can produce logical and credible predictions. However, overly prioritizing accuracy over interpretability may lead to numerous unrealistic rules that deviate from actual conditions. To overcome this problem, the belief distribution for each rule should be restricted, as shown in Formula (8).

8 $$\begin{aligned} \begin{aligned} U_\beta :\{&\left\{ \beta _1 \le \beta _2 \le \ldots \le \beta _n\right\} \\&\text{ or } \left\{ \beta _1 \ge \beta _2 \ge \ldots \ge \beta _n\right\} \\&\text{ or } \left\{ \beta _1 \le \ldots \le \max \left( \beta _1, \beta _2, \ldots , \beta _n\right) \ge \ldots \ge \beta _n\right\} \end{aligned} \end{aligned}$$

where $U_\beta$ denotes the constraints on the belief distribution. In the IBRB-m based student performance prediction model, students are evaluated in five grades: Excellent, Good, Pass, Fair, and Fail. Assuming the student’s assessment is (Excellent, 0.35), (Good, 0.25), (Pass, 0.2), (Fair, 0.15), (Fail, 0.05). However, the optimized belief distribution may be (Excellent, 0.4), (Good, 0.1), (Pass, 0), (Fair, 0.1), (Fail, 0.4), and if the belief distribution takes a concave shape, this means that the assessment results are in both excellent and failing states, which is not reasonable in the real world. A reasonable shape for the belief distribution should be monotonic or convex. This is shown in Fig. 3.

The proposed interpretability optimization criteria are selected based on a synthesis of established interpretable AI principles and the specific requirements for trustworthy educational prediction. Criterion 1 prioritizes the integration of expert knowledge as the initialization anchor, ensuring the model starts from a pedagogically meaningful basis . Criterion 2 constrains parameter drift to prevent the optimized model from deviating into a counter-intuitive “grey box,” thereby preserving the link between model parameters and real-world concepts essential for educator trust. Criterion 3 allows the adaptive refinement of reference values within expert-defined bounds, balancing data-driven accuracy with semantic consistency. Finally, Criterion 4 enforces plausible belief distribution shapes (e.g., monotonic or convex) to ensure that the model’s probabilistic outputs reflect realistic student performance states, enhancing both face validity and practical utility. Together, these criteria form a coherent framework that guides the optimization process to maintain a transparent, accurate, and educationally sound prediction model.

### Definition of the structure of the prediction model

The student performance prediction model based on IBRB-m consists of a series of belief rules. The description of the $k_{t h}$ rule is shown in Formula (9).

9 $$\begin{aligned} \begin{aligned}&BeliefRule_k:\\&If\; \textrm{x}_1\; is\; \textrm{A}_1 \wedge x_2\; is\; \textrm{A}_2 \wedge \ldots \wedge x_M\; is\; \textrm{A}_M\\&Then\; result\; is \left\{ \left( \textrm{H}_1, \beta _1\right) ,\left( \textrm{H}_2, \beta _2\right) , \ldots ,\left( \textrm{H}_N, \beta _N\right) \right\} \\&with\; rule\; weight \theta _1, \theta _2, \ldots , \theta _k\\&and \;attribute\; weight \delta _1, \delta _2, \ldots , \delta _M\\ \end{aligned} \end{aligned}$$

where $x_i(i=1, \ldots , M)$ refers to the student performance predictors; $A_i(i=1, \ldots , M)$ denotes the set of reference values of the predictors; $\textrm{H}_{\textrm{i}}(i=1, \ldots , N)$ is the performance prediction results of the model; $\beta _{\textrm{i}}(i=1, \ldots , N)$ denotes the belief level corresponding to each result; $\theta _i(i=1, \ldots , k)$ denotes the rule weight of the *i* belief rule; $\delta _1(i=1, \ldots , M)$ denotes the attribute weight of the *i* predictor; *M* denotes the number of predictors.

### Reasoning process for the prediction model

The inference process of the IBRB-m based model is executed using the ER algorithm. Following traditional BRB interpretability criteria 6–7 in Sect. 3.2, the reasoning steps can be summarized as follows.

First, the match between input data and reference values is computed, as shown in Eq. (10).

10 $$\begin{aligned} \begin{aligned}&S\left( x_i\right) =\left\{ \left( A_{i, j}^k \alpha _{i, j}^k\right) \mid i=1, \ldots , T_k, j=1, \ldots , J_i\right\} \\&\alpha _{i, j}^k= {\left\{ \begin{array}{ll}\frac{A_{i, j+1}^k-x_i}{A_{A_{i, j+1}^k}^k-A_{i, j}^k} & , j=k\left( A_{i, j}^k \le x_i \le A_{i, j+1}^k\right) \\ \frac{x_i-A_{i, j}^k}{A_{i, j+1}^k-A_{i, j}^k} & , j=k+1 \\ 0 & , j=1, \ldots , J_i(j \ne k, k+1)\end{array}\right. } \end{aligned} \end{aligned}$$

where $S\left( x_i\right)$ represents the transformation process of input data $x_i$; $\alpha _{i, j}^k$ denotes the matching degree of $x_i$ for the $j_{t h}$ reference value belonging to the $i_{t h}$ attribute; $J_i$ denotes the number of reference values; $A_{i, j}^k$ and $A_{i, j+1}^k$ denote two adjacent reference values.

After matching, activation weights are calculated, as shown in Eq. (11).

11 $$\begin{aligned} \omega _{k} = \frac{\theta _{k} \prod _{i=1}^{T_{k}} \left( a^{k}_{i,j} \right) ^{\bar{\delta }_i}}{\sum _{l=1}^{L} \theta _{l} \prod _{i=1}^{T_{k}} \left( a^{l}_{i,j} \right) ^{\bar{\delta }_i}}, \quad \bar{\delta }_i = \frac{\delta _i}{\max _{i} \delta _i} \end{aligned}$$

where $\omega _k$ denotes the activation weight of the rule $k_{t h}$; $\overline{\delta _i}$ denotes the relative weight of the attribute.

Subsequently, the ER parsing algorithm is utilized to determine the credibility of the results. The specific inference steps can be referred to in Eq. (12).

12 $$\begin{aligned} \begin{gathered} \beta _n=\frac{\mu \left[ \prod _{k=1}^L\left( \omega _k \beta _{n, k}+1-\omega _k \sum _{j=1}^N \beta _{j, k}\right) -\prod _{k=1}^L\left( 1-\omega _k \sum _{j=1}^N \beta _{j, k}\right) \right] }{1-\mu \left[ \prod _{k=1}^L\left( 1-\omega _k\right) \right] } \\ \mu =\left[ \sum _{n=1}^N \prod _{k=1}^L\left( \omega _k \beta _{n, k}+1-\omega _k \sum _{j=1}^N \beta _{j, k}\right) -(N-1) \prod _{k=1}^L\left( 1-\omega _k \sum _{j=1}^N \beta _{j, k}\right) \right] ^{-1} \end{gathered} \end{aligned}$$

where $\beta _n(\textrm{n}=1,2, \ldots , \mathrm {~N})$ represents the belief level of the $n_{t h}$ result $D_n$, the output of IBRB-m based model is shown in Eq. (13).

13 $$\begin{aligned} S(\tilde{x})=\left\{ \left( D_n, \beta _n\right) \mid n=1,2, \ldots , N\right\} \end{aligned}$$

where $S(\tilde{x})$ denotes the belief distribution obtained from the set of data $\tilde{x}$ of the attribute. Finally, the inference results based on the IBRB-m model were calculated. As shown in Eq. (14).

14 $$\begin{aligned} u(S(\tilde{x}))=\sum _{n=1}^N u\left( D_n\right) \beta _n \end{aligned}$$

where $u(S(\tilde{x}))$ denotes the final outcome of the IBRB-m based model. $\textrm{u}\left( D_n\right)$ denotes the utility value of the $n_{t h}$ outcome level. Therefore, the prediction result of the IBRB-m based model is denoted as *u*(*S*(*x*)).

### A new P-CMA-ES algorithm

This study adopts the improved P-CMA-ES algorithm. By introducing the knowledge of all domain experts, including belief level, reference values, rule weights, and attribute weights, reasonable constraints are imposed on the parameter space to ensure that all parameters are optimized within a reasonable range. While maintaining the interpretability of the model, the modeling accuracy is significantly improved. This algorithm effectively avoids the “black box” problem through the constraints guided by expert knowledge, making the optimized parameters both theoretically interpretable and in line with practical application requirements. The experimental results show that this method of embedding domain knowledge into the optimization process can ensure computational stability while taking into account the improvement of prediction performance and the maintenance of model interpretability. It is particularly suitable for educational modeling scenarios such as student performance prediction that require a balance between data-driven performance and theoretical rationality.

The improved P-CMA-ES algorithm introduces two important modifications on the basis of the original P-CMA-ES and its standard variant based on projection, fundamentally altering its role from a general-purpose optimizer to an interpretability-preserving one. First, the set of optimized parameters is extended to include the reference values of the input attributes, which are typically kept fixed in traditional belief rule base optimization. This allows the model to data-adaptively refine the semantic partitions of the input space. Second, and more critically, the optimization process is governed by a set of explicit. These constraints, derived from the proposed interpretability criteria, ensure that the optimization respects expert knowledge by bounding parameter drift and enforcing plausible belief distribution shapes (e.g., monotonic or convex). Consequently, the core difference lies not in the evolutionary mechanism itself, but in the integration of domain knowledge directly into the parameter set and the constraint landscape, ensuring the search for high accuracy does not compromise the model’s transparency. To clearly illustrate this improved optimization process, Algorithm 2 presents the pseudocode of the modified P-CMA-ES algorithm with interpretability constraints.

**Figure:**
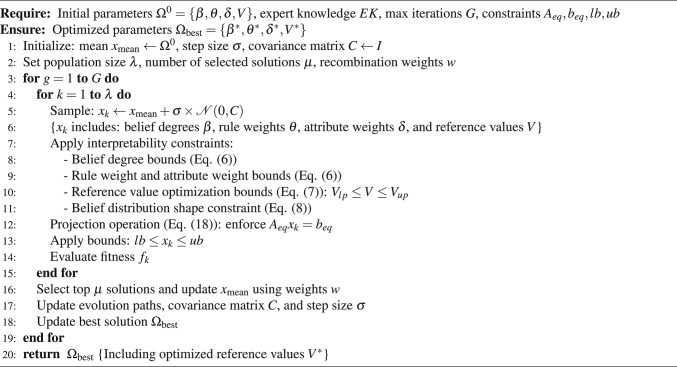
Algorithm 2 Improved P-CMA-ES with reference value optimization

**Fig. 4:**
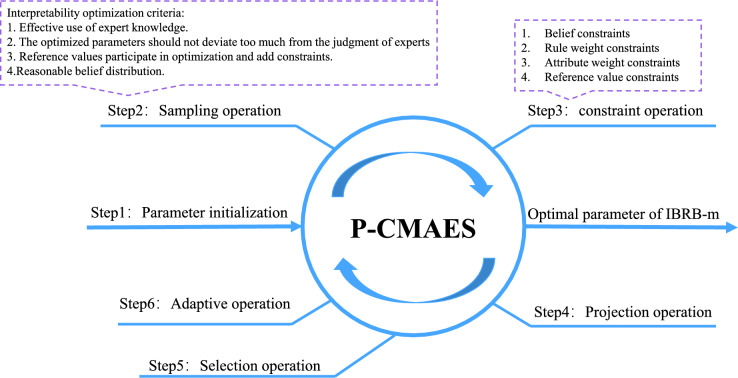
The optimization process of the IBRB-m model.

The improved P-CMA-ES algorithm is shown in Fig. 4 and the specific steps of optimization are as follows:

Step 1 (Initialization operation): In addition to the belief, rule weights, and attribute weights to be optimized, the interpretability optimization criteria 3 also includes the reference values in the set of parameters to be optimized. The set of parameters to be optimized is shown in Eq. (15).

15 $$\begin{aligned} \Omega ^0=\left\{ \beta _{1,1}, \ldots , \beta _{N, k}, \theta _1, \ldots , \theta _k, \delta _1, \ldots , \delta _M, V_1, \ldots , V_c\right\} \end{aligned}$$

Step 2 (Sampling operation): The interpretability optimization criteria 1 ensures initial interpretability by translating expert knowledge into parameters for the initial population. The actions to be performed are shown in Eq. (16).

16 $$\begin{aligned} \Omega _s^{g+1}=u^g+o^g \varepsilon \left( 0, M^g\right) , s=1, \ldots , h \end{aligned}$$

where $\Omega _s^{g+1}$ is the $S_{t h}$ solution in the $(g+1)_{t h}$ generation.$u^g$ represents the mean of the population in the $g_{t h}$ generation.$\boldsymbol{o}^g$ represents the step size. $\varepsilon (\cdot )$ represents the normal distribution.$M^g$ represents the covariance matrix.

Step 3 (Constraint operation): According to the interpretability optimization criteria 2 and 4, constraints are added to the expert knowledge to ensure that the model optimization process is based on the local judgments of the experts. This ensures that the optimized parameters maintain a high degree of similarity with the expert knowledge. The constraint limitations can be described as Eq. (17).

17 $$\begin{aligned} p_{9,10}=\left\{ \begin{array}{cc} \theta _{l p_k} \le \theta _k \le \theta _{u p_k} & \textrm{k} \in \{1,2, \ldots , \mathrm {~L}\}, \\ \delta _{l p_i} \le \delta _i \le \delta _{u p_i} & \textrm{n} \in \{1, \ldots , N\}. \\ \beta _{l p_{k, n}} \le \beta _{k, n} \le \beta _{u p_{k, n}} & \textrm{i}, \mathrm {~m} \in \left\{ 1,2, \ldots , T_k\right\} . \\ V_{l p_u}<V_u<V_{u p_u} & \textrm{u} \in \{1,2, \ldots , \textrm{Z}\}. \end{array}\right. \end{aligned}$$

Introducing reference values into the optimization process not only provides more specific benchmarks for the optimization goals and ensures that the optimization results are more closely aligned with the actual requirements, but also improves the transparency of the optimization process by introducing interpretable constraints.

Step 4 (Projection operation): The projection operation maps the solution to a valid range within the constraint-defined hyperplane, as shown in Eq. (18).

18 $$\begin{aligned} \begin{aligned}&\Omega _s^{g+1}(1+j \times (\psi -1): j \times \psi ) \\&=\Omega _s^{g+1}(1+j \times (\psi -1): j \times \psi )-S^G \times \left( S \times S^G\right) ^{-1} \\&\times \Omega _s^{g+1}(1+j \times (\psi -1): j \times \psi ) \times S \end{aligned} \end{aligned}$$

where *j* denotes the number of constraint variables,$\psi$ denotes the number of design constraints, and *S* denotes the number of parameters.

Step 5 (Selection operation): Sort the population using Formula (19), select the next generation’s optimal solution, and adjust the mean value.

19 $$\begin{aligned} u^{g+1}=\sum _{s=1}^\eta z_s \Omega _{s: \lambda }^{g+1} \end{aligned}$$

where $Z_s$ refers to the coefficients used in the weighted reorganization.

Step 6 (Adaptive operation): Update the covariance matrix and step size to steer population evolution, as shown in Eqs. (20) and (21).

20 $$\begin{aligned} \begin{aligned}&C^{g+1}=\left( 1-c_1-c_2\right) C^g+c_1 p_c^{g+1}\left( p_c^{g+1}\right) ^T \\&+c_s \sum _{\phi =1}^\omega \tau _g\left( \frac{\varphi _{\phi : \omega }^{g+1}-o^g}{\gamma ^g}\right) \left( \frac{\varphi _{\phi : \omega }^{g+1}-o^g}{\gamma ^g}\right) ^T \end{aligned} \end{aligned}$$

21 $$\begin{aligned} p_e^{g+1}=\left( 1-c_e\right) \times p_e^g+\sqrt{c_e\left( 2-c_e\right) \left( \sum _{\phi =1}^\omega q_\phi ^2\right) ^{-1}} \times \frac{o^{g+1}-o^g}{\gamma ^g} \end{aligned}$$

where $c_1, c_2, c_s, c_e$ denote the learning rate.

## Case studies

In this section, a student achievement dataset is used as an example to test the validity of the IBRB-m based model. In Sect. 4.1, an introduction to the dataset and attribute selection is provided. In Sect. 4.2, a student performance prediction model based on IBRB-m is developed. The ablation experiment is described in Sect. 4.3. Cross-validation is performed in Sect. 4.4. A comparison experiment is conducted in Sect. 4.5. Statistical significance analysis was conducted in Sect. 4.6. Sect. 4.7 discusses the practical significance of model interpretability for educators.

### Data set and attribute selection

The dataset available at https://www.kaggle.com/datasets/rabieelkharoua/students-performance-dataset is used in this study. The dataset provides thirteen factors that influence student performance. The features encompass three main categories: academic and behavioral indicators (GPA, weekly study time, number of absences), demographic and background information (age, gender, ethnicity, parental education level), and support and extracurricular factors (parental support, tutoring, participation in sports, music, volunteering, and overall extracurricular engagement level). Student performance was ultimately assessed on a five-point scale from 0 to 4. This dataset contains 2392 data points, of which 1675 were randomly selected for training and 717 for testing.

The target variable is the student’s final performance grade, which is mapped to a five-level ordinal scale: 0 (Excellent), 1 (Good), 2 (Pass), 3 (Fair), and 4 (Fail). The distribution across these performance levels is moderately balanced. The GPA scores range from 1.2 to 4.0 with a mean of 2.85, absences range from 0 to 18 with a mean of 3.2, and weekly study time ranges from 1 to 10 hours with a mean of 5.4 hours.

After the importance ranking by the method described in Sect. 3.2, each attribute was strictly ranked according to the obtained results, which allowed the identification of the most critical attributes contributing to the model performance. After screening and eliminating the attributes with low importance, the specific importance ranking is shown in Table 1:

**Table 1:** Ranking of importance.

Attribute	Attribute importance
GPA	0.5028
Absences	0.2311
StudyTime Weekly	0.0804
Parental Support	0.0328
Parental Education	0.0285
Age	0.0269
Ethnicity	0.0251
Gender	0.0137
Tutoring	0.0134
Sports	0.0133
Extracurricular	0.0132
Music	0.0098
Volunteering	0.0089

In the analysis of the dataset, it is evident that the attributes GPA (Grade Point Average) and Absences (number of absences) have a very high percentage of importance in the overall dataset, accounting for 73%. The significant difference in their importance compared to other attributes indicates a strong association and influence between these two attributes and the overall characteristics of the dataset. Therefore, from the perspective of statistical analysis and model construction, it is reasonable to choose these two attributes, GPA and Absences, to represent the whole dataset. This is mainly because they reflect most of the volatility or changes in the dataset well, and thus these two attributes should be prioritized in the subsequent modeling or decision-making process.

**Fig. 5:**
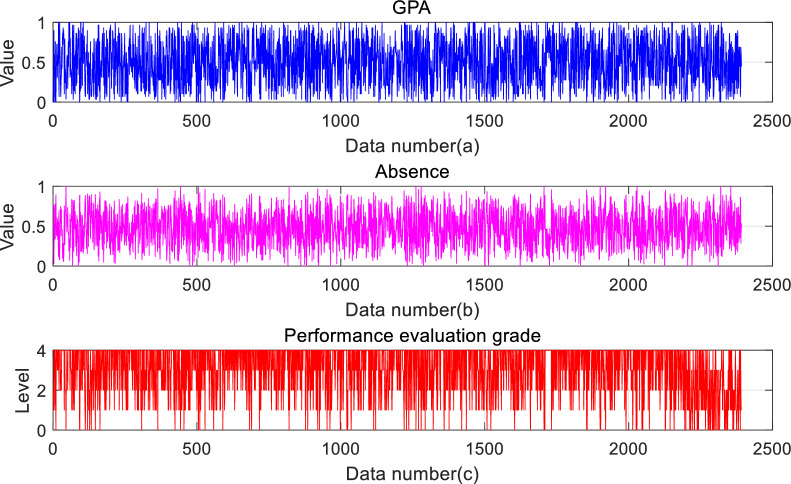
Data distribution.

Since the numerical magnitude of each indicator is different, data standardization is essential. This is done to ensure that all data are within the uniform range of [0,1]. Since this experiment aims to solve a classification problem, the resultant attributes are not involved in the standardization. The standardization formula is shown in Eq. (22), and the processed data are shown in Fig. 5a and b.

22 $$\begin{aligned} \tilde{x}=\frac{x-x_{\min }}{x_{\max }-x_{\min }} \end{aligned}$$

The selection of GPA and Absences as the final feature set results from a deliberate trade-off between predictive power, model complexity, and educational interpretability. While alternative feature selection methods exist–such as mutual information, correlation-based filters, or wrapper methods like recursive feature elimination–the Random Forest based approach was chosen for its resilience to multicollinearity and its ability to evaluate feature importance through non-linear, ensemble-based permutations. In preliminary tests comparing methods, RF provided the most stable importance rankings across bootstrap samples, which is crucial for building a reliable model.

The decision to retain only the top two features, despite the cumulative importance of subsequent features, is primarily motivated by the interpretability-simplicity principle central to our IBRB-m framework. Each additional attribute exponentially increases the number of belief rules, directly conflicting with our goal of avoiding combinatorial explosion. Furthermore, from a pedagogical standpoint, GPA and Absences represent the most direct and universally available indicators for instructors: GPA encapsulates prior academic achievement, while Absences is a tangible, early-warning behavioral metric. This parsimonious selection ensures the model remains not only accurate but also practically actionable and easily explainable in real educational settings.

### Construction and optimization of a predictive model of student performance based on IBRB-m

The initial model based on IBRB-m needs to establish belief rules, the construction is shown in Formula (23).

23 $$\begin{aligned} \begin{aligned}&\text{ If } \textrm{x}_1 \text{ is } \textrm{A}_1 \wedge x_2 \text{ is } \textrm{A}_2\\&\text{ Then } \text{ result } \text{ is } \left\{ \left( \textrm{H}_1, \beta _1\right) ,\left( \textrm{H}_2, \beta _2\right) ,\left( \textrm{H}_3, \beta _3\right) ,\left( \textrm{H}_4, \beta _4\right) ,\left( \textrm{H}_5, \beta _5\right) \right\} \\&\text{ with } \text{ rule } \text{ weight } \theta _1, \theta _2, \ldots , \theta _k\\&\text{ and } \text{ attribute } \text{ weight } \delta _1, \delta _2\\ \end{aligned} \end{aligned}$$

where $\textbf{x}_1$ represents the number of absences per week,$\textbf{x}_2$ represents the GPA;H represents the final performance grade. In this experiment, the grades are categorized into five levels: excellent (E), good (G), qualified (Q), average (A), and unqualified (U). These levels are denoted by the numbers 0 to 4 in the model, respectively.

Based on expert knowledge and in-depth analysis of indicator data, 5 reference values were set for each indicator, and 25 belief rules were constructed. The sum of the matches between the selected reference values and the data of the current input indicators was limited to the range from 0 to 1. The experiment uses five indicators, numbered from 0 to 4, to assess the performance level of the students, with 0 representing excellent, 1 representing good, and so on. The initial reference points and reference value settings for the input metrics and output results are shown in Table 2. The IBRB-m was built by integrating domain expertise, and the initial rule settings are given in Table 3.

**Table 2:** The initial weight of attributes and reference values.

Indicator	E	G	Q	A	U	Attribute weight	constraint
$x_1$	0	0.2	0.4	0.7	1	1	0.6–1
$x_2$	0	0.25	0.5	0.75	1	1	0.6–1

**Table 3:** The initial rules of the IBRB-m.

$x_1 \wedge x_2$	Rule weigh $\theta _k$	The initial belief	The belief constraint
$E \wedge E$	0.9	{0.9, 0.1, 0, 0, 0}	{0.8-1, 0-0.2, 0-0.1, 0-0.1,0-0.1}
$E \wedge G$	0.9	{0.3, 0.35, 0.15, 0.1, 0.1}	{0.2-0.4, 0.3-0.4, 0.1-0.2, 0-0.2, 0-0.2}
$E \wedge Q$	0.9	{0.15, 0.15, 0.15, 0.15, 0.4}	{0.1-0.2, 0.1-0.2, 0.1-0.2, 0.1-0.2, 0.3-0.5}
$E \wedge A$	0.9	{0.1, 0.1, 0.1, 0.5, 0.2}	{0-0.2, 0-0.2, 0-0.2, 0.4-0.6, 0.1-0.3}
$E \wedge U$	0.9	{0.1, 0.4, 0.3, 0.1, 0.1}	{0-0.2, 0.3-0.5, 0.2-0.4, 0-0.2, 0-0.2}
$G \wedge E$	0.9	{0.15, 0.15, 0.4, 0.15, 0.15}	{0.1-0.2, 0.1-0.2, 0.3-0.5, 0.1-0.2, 0.1-0.2}
$G \wedge G$	0.9	{0.15, 0.4, 0.15, 0.15, 0.15}	{0.1-0.2, 0.3-0.5, 0.1-0.2, 0.1-0.2, 0.1-0.2}
$G \wedge Q$	0.9	{0.35, 0.3, 0.15, 0.1, 0.1}	{0.3-0.4, 0.2-0.4, 0.1-0.2, 0-0.2, 0-0.2}
$G \wedge A$	0.9	{0.1, 0.1, 0.3, 0.4, 0.1}	{0-0.2, 0-0.2, 0.2-0.4, 0.3-0.5, 0-0.2}
$G \wedge U$	0.9	{0.1, 0.4, 0.2, 0.2, 0.1}	{0-0.2, 0.3-0.5, 0.1-0.3,0.1-0.3,0-0.2}
$Q \wedge E$	0.9	{0.1, 0.3, 0.3, 0.15, 0.15}	{0-0.2, 0.2-0.4, 0.2-0.4, 0.1-0.2, 0.1-0.2}
$Q \wedge G$	0.9	{0.4, 0.15, 0.15, 0.15, 0.15}	{0.3-0.5, 0.1-0.2, 0.1-0.2, 0.1-0.2, 0.1-0.2}
$Q \wedge Q$	0.9	{0.4, 0.15, 0.15, 0.15, 0.15}	{0.3-0.5, 0.1-0.2, 0.1-0.2, 0.1-0.2, 0.1-0.2}
$Q \wedge A$	0.9	{0.1, 0.1, 0.1, 0.4, 0.3}	{0.0-2, 0-0.2, 0-0.2, 0.3-0.5, 0.2-0.4}
$Q \wedge U$	0.9	{0.1, 0.15, 0.15, 0.3, 0.3}	{0-0.2, 0.1-0.2, 0.1-0.2, 0.2-0.4, 0.2-0.4}
$A \wedge E$	0.9	{0.1, 0.25, 0.25, 0.2, 0.2}	{0-0.2, 0.2-0.3, 0.2-0.3, 0.1-0.3, 0.1-0.3}
$A \wedge G$	0.9	{0.2, 0.2, 0.4, 0.1, 0.1}	{0.1-0.3, 0.1-0.3, 0.3-0.5, 0-0.2, 0-0.2}
$A \wedge Q$	0.9	{0.35, 0.35, 0.1, 0.1, 0.1}	{0.3-0.4, 0.3-0.4, 0-0.2, 0-0.2, 0-0.2}
$A \wedge A$	0.9	{0, 0, 0.3, 0.3, 0.4}	{0.0-1, 0-0.1, 0.2-0.4, 0.2-0.4, 0.3-0.5}
$A \wedge U$	0.9	{0, 0.2, 0.2, 0.2, 0.4}	{0-0.1, 0.1-0.3, 0.1-0.3, 0.1-0.3, 0.3-0.5}
$U \wedge E$	0.9	{0.4, 0.3, 0.1, 0.1, 0.1}	{0.3-0.5, 0.2-0.4, 0-0.2, 0-0.2, 0-0.2}
$U \wedge G$	0.9	{0.25, 0.3, 0.15, 0.15, 0.15}	{0.2-0.3, 0.2-0.4, 0.1-0.2, 0.1-0.2, 0.1-0.2}
$U \wedge Q$	0.9	{0.3, 0.25, 0.15, 0.15, 0.15}	{0.2-0.4, 0.2-0.3, 0.1-0.2, 0.1-0.2, 0.1-0.2}
$U \wedge A$	0.9	{0, 0, 0.3, 0.3, 0.4}	{0-0.1, 0-0.1, 0.2-0.4, 0.2-0.4, 0.3-0.5}
$U \wedge U$	0.9	{0, 0, 0, 0.1, 0.9}	{0-0.1, 0.0-1, 0.0-1, 0-0.2, 0.8-1}

The input attributes of this experiment are GPA and Absences, corresponding to $\textbf{x}_1$ and $\textbf{x}_2$ respectively, and H is the model prediction outcome. The initial model is optimized with 400 iterations and a total of 2392 data points collected. Expert knowledge, which is the summary of students’ long-term performance, is an significant interpretable element in the student performance prediction model based on IBRB-m. Utilizing expert knowledge to set the initial belief distribution, The model fine tunes the initial belief interval defined by experts through optimization algorithms, ensuring that the final output belief interval does not significantly deviate from the expert’s initial setting. The interpretability of the model is stronger when its output belief interval is closer to the initial interval; that is, the results of the model are more easily understood and accepted by users. Table 4 presents the optimized reference values and attribute weights. Table 5 presents the optimized rules.

**Table 4:** The optimized reference values.

Indicator	E	G	Q	A	U	Attribute weight
$x_1$	0	0.1863	0.3223	0.6728	1	0.70
$x_2$	0	0.3209	0.5815	0.8349	1	0.95

**Table 5:** The optimized rules.

No.	$x_1 \wedge x_2$	Rule weigh $\theta _k$	The optimized rules
1	$E \wedge E$	0.819	{0.9495,0.0517,0,0,0}
2	$E \wedge G$	0.665	{0.3091,0.3580,0.2077,0.0846,0.0406}
3	$E \wedge Q$	0.698	{0.1601,0.2392,0.3780,0.1320,0.0908}
4	$E \wedge A$	0.826	{0.060,0.102,0.110,0.470,0.2570}
5	$E \wedge U$	0.713	{0.0410,0.4683,0.3004,0.1251,0.0652}
6	$G \wedge E$	0.531	{0.1015,0.1332,0.3738,0.2026,0.1889}
7	$G \wedge G$	0.923	{0.2168,0.4155,0.1622,0.0904,0.1151}
8	$G \wedge Q$	0.960	{0.4010,0.2755,0.1599,0.0900,0.0737}
9	$G \wedge A$	0.732	{0.0243,0.0831,0.2294,0.4861,0.1771}
10	$G \wedge U$	0.624	{0.0476,0.3492,0.2685,0.2547,0.080}
11	$Q \wedge E$	0.527	{0.1177,0.2151,0.3111,0.2011,0.1550}
12	$Q \wedge G$	0.657	{0.3523,0.2388,0.2106,0.1418,0.0565}
13	$Q \wedge Q$	0.585	{0.4874,0.1408,0.1359,0.1331,0.1028}
14	$Q \wedge A$	0.956	{0.0244,0.0450,0.1021,0.4510,0.3775}
15	$Q \wedge U$	0.585	{0.0776,0.0845,0.1680,0.3460,0.3238}
16	$A \wedge E$	0.802	{0.1624,0.1631,0.3328,0.1757,0.1659}
17	$A \wedge G$	0.932	{0.1908,0.2841,0.3667,0.1100,0.0484}
18	$A \wedge Q$	0.611	{0.2841,0.2744,0.1846,0.1326,0.1243}
19	$A \wedge A$	0.635	{0,0,0.3336,0.3251,0.3402}
20	$A \wedge U$	0.612	{0,0.1095,0.2111,0.2740,0.4052}
21	$U \wedge E$	0.630	{0.4465,0.2614,0.1600,0.1008,0.0313}
22	$U \wedge G$	0.596	{0.2736,0.3114,0.1567,0.1359,0.1224}
23	$U \wedge Q$	0.926	{0.3312,0.2387,0.1578,0.1507,0.1216}
24	$U \wedge A$	0.652	{0,0.0008,0.2645,0.2993,0.4359}
25	$U \wedge U$	0.708	{0,0,0,0.1125,0.8859}

As shown in Tables 2 and 4, the spatial division intervals of reference values before and after optimization are different from each other and do not overlap, which meets traditional interpretability criterion 1. The optimized rules are shown in Table 5, the data distribution is convex or monotonic, meeting interpretability optimization criteria 4.

### Ablation experiments

#### Precision analysis

To clearly demonstrate the modular contributions, this study designs a progressive ablation experiment that incrementally introduces the core components proposed in this paper. Specifically, the following three models are constructed:

- Benchmark Model (BRB): This model is constructed based on initial expert knowledge, without introducing any interpretability criteria, and employs the standard PCMAES algorithm to optimize a subset of parameters, representing the traditional approach.
- Contrastive Model (BRBI): Building upon BRB, this model incorporates traditional interpretability criteria and adheres to the interpretability optimization criteria 1, 2, and 4 proposed in this paper. However, its set of optimized parameters remains the same as that of BRB. It is intended to independently evaluate the impact of introducing these new partial interpretability criteria per se.
- Complete Model Proposed in This Paper (IBRB-m): Based on BRBI, this model further adopts all interpretability criteria proposed in this paper along with the novel multi-parameter optimization algorithm (improved P-CMA-ES). The essential enhancement of this algorithm lies in incorporating reference values into the optimization parameter set and performing joint optimization under the constraints of the new criteria. Therefore, the core difference between IBRB-m and BRBI resides in the upgrade of the optimization algorithm and the expansion of the optimization scope.

**Fig. 6:**
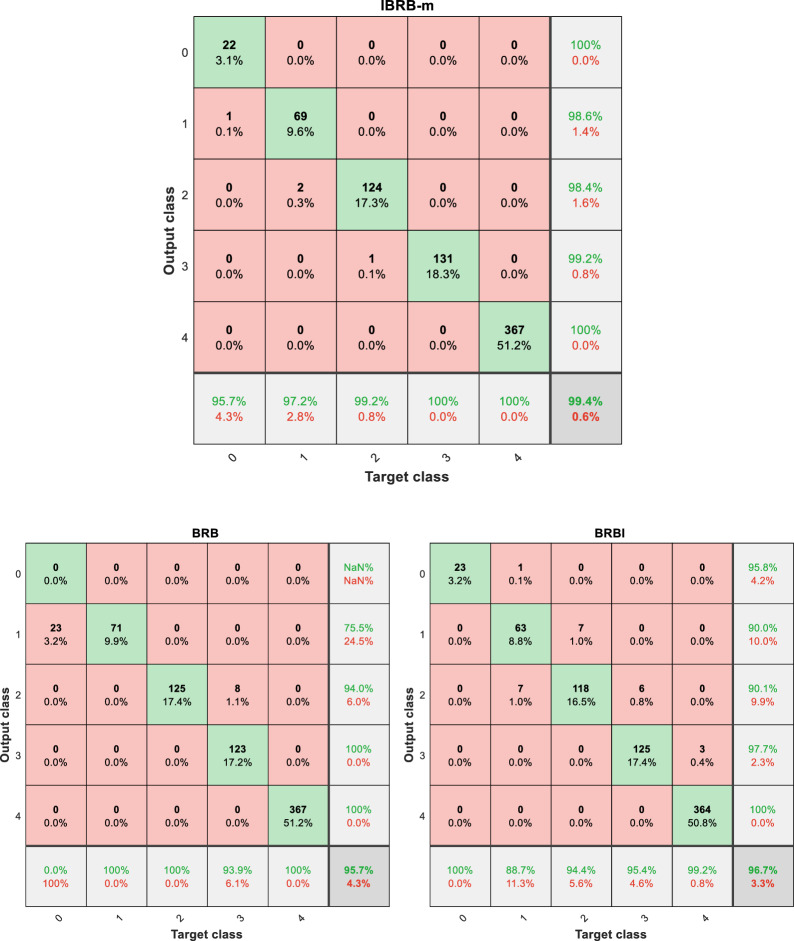
Comparison of different models.

The comparison results are shown in Fig. 6. The accuracy of the IBRB-m based model is 99.4%, while the accuracies of the BRB-based and BRBI-based models are 95.7% and 96.7%, respectively. This is mainly because the interpretability criterion proposed in this paper fully utilizes expert knowledge in the modeling process. Given that the reference value, as a vital component of the expert knowledge, substantially affects the model’s prediction accuracy, the introduced reference value optimization strategy ensures the validity of the reference value to a large extent. This method significantly improves the overall performance and prediction accuracy of the model.

**Fig. 7:**
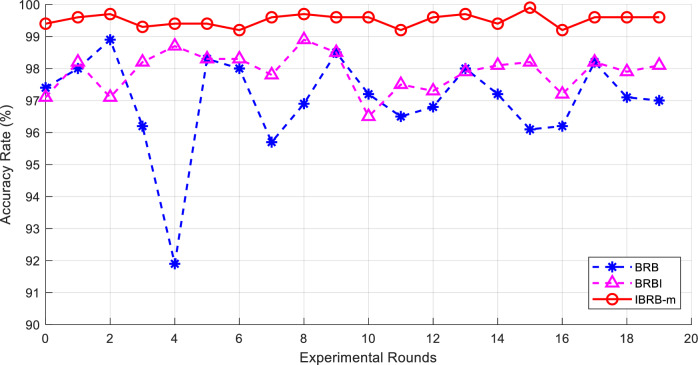
Comparison chart of accuracy rates for the first group of experiments.

In order to further confirm the effectiveness of the model, all methods were repeated twenty times, and the average of the results of twenty independent experiments was taken to more accurately reflect the overall performance and stability of the model. The experimental results are shown in Table 6. In comparison with the traditional BRB, the accuracy of the IBRB-m based model is enhanced by 2.51%. In comparison with the BRBI model, the accuracy is enhanced by 1.62%. This set of experiments verifies the excellence of the IBRB-m based model with regard to accuracy and proves that the improvement of the model is effective. Figure 7 provides a visualization graph of the experimental results.

**Table 6:** Model accuracy comparison.

Model	Highest accuracy (%)	Minimum accuracy (%)	Average accuracy (%)
BRB	98.9	91.9	97.005
BRBI	98.9	96.5	97.9
IBRB-m	99.9	99.2	99.515

#### Interpretability analysis

Euclidean distance serves as a mathematical method to measure the distance between two points in a multidimensional space. The computation is based on the square root of the sum of the squares of the differences in coordinates across each dimension. It is widely used in machine learning and data analysis to evaluate the similarity or distance between data points. Characterized by being intuitive and easy to calculate, Euclidean distance is a popular choice. In this paper, the Euclidean distance is chosen as an index for evaluating the interpretability of the model, and its calculation is shown in Formula (24). The Euclidean distances of the traditional BRB-based and BRBI-based models are 1.4792 and 0.5032, respectively, while that of the IBRB-m based model is 0.2916. It can be seen that, in terms of interpretability, the IBRB-m based model shows significant advantages.

**Fig. 8:**
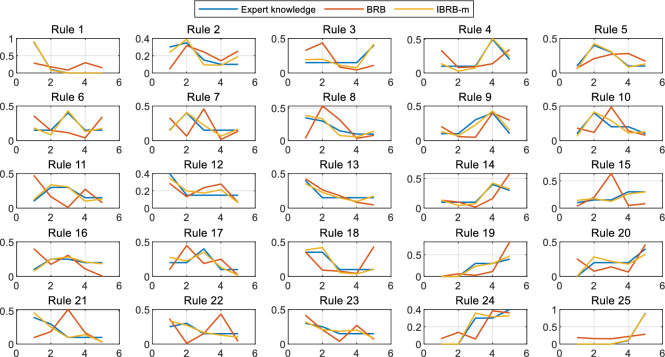
Belief distribution for each rule for BRB and IBRB-m.

The comparison of each belief distribution in the student performance prediction model based on BRB and IBRB-m is shown in Fig. 8. In this figure, the belief distribution of IBRB-m based model is consistent with the expert knowledge, demonstrating that the model’s interpretability is assured and the optimized rules are trustworthy. On the other hand, the BRB based model has completely deviated from the expert’s initial judgment during the rule optimization process. This deviation results in the optimized model structure lacking the original model’s inherent interpretability properties.

**Fig. 9:**
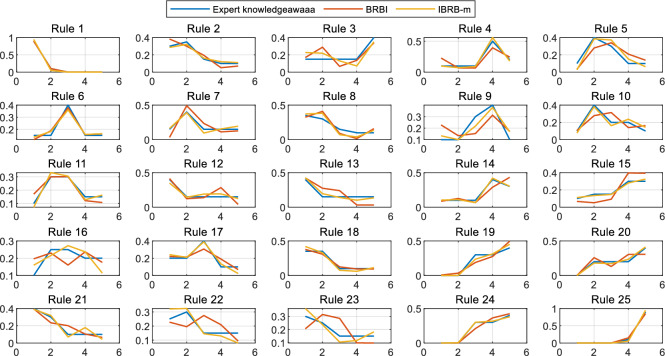
Belief distribution for each rule for BRBI and IBRB-m.

The comparison of each belief distribution of the student performance prediction model based on BRBI and IBRB-m is shown in Fig. 9. When comparing the performance of the two models, both of which are interpretable, it is observed that the trend of the belief distributions of both is in line with the expert knowledge. However, by carefully analyzing Rules 2, 4, 7, 10, 13, 14, and 15, it can be observed that the performance of the IBRB-m model on these rules is more compatible with expert knowledge. Combined with the metric of Euclidean distance, it can be concluded that the IBRB-m based model achieves further improvement in the interpretability dimension compared to the BRBI based model.

24 $$\begin{aligned} \rho \left( \textrm{h}_n, \mathrm {~h}_n^{\prime }\right) =\sqrt{\left( h_1-h_1^{\prime }\right) ^2+\left( h_2-h_2^{\prime }\right) ^2+\ldots +\left( h_{\textrm{n}}-h_{\textrm{n}}^{\prime }\right) ^2}=\sqrt{\sum _{\textrm{i}=1}^{\textrm{n}}\left( h_i-h_i^{\prime }\right) ^2} \end{aligned}$$

where $h_i$ is the expert knowledge, $h_i^{\prime }$ is the optimized parameters and $\rho \left( \textrm{h}_n, \mathrm {~h}_n^{\prime }\right)$ is the Euclidean distance between the expert knowledge and the optimized parameters.

### Cross-validation

To ensure robust evaluation of model performance and prevent overfitting, we employed *k*-fold cross-validation throughout our experiments. Cross-validation is essential for assessing the generalization ability of predictive models on unseen data and provides a more reliable estimate of model performance compared to a single train-test split.

Specifically, we implemented 5-fold cross-validation on the complete dataset containing 2392 student records. The data was randomly partitioned into five mutually exclusive subsets of approximately equal size, with each fold containing about 20% of the data.

In each of the five iterations, one fold was designated as the test set, while the remaining four folds constituted the training set. This process ensured that every data point was used exactly once for testing, providing a comprehensive assessment of model performance across the entire dataset. Importantly, all model development steps–including feature selection, parameter initialization based on expert knowledge, and optimization using the improved P-CMA-ES algorithm–were performed independently within each training fold to prevent data leakage.

The cross-validation results for the proposed IBRB-m model and two baseline models are presented in Table 7. The performance metrics for each fold and the average across all folds are reported.

**Table 7:** Cross-validation results (Accuracy) for different models.

Model	Fold 1 (%)	Fold 2 (%)	Fold 3 (%)	Fold 4 (%)	Fold 5 (%)	Average (%)
BRB	98.3	96.4	96.0	98.5	96.1	97.06
BRBI	98.7	96.9	96.2	97.9	93.9	96.72
IBRB-m	99.4	99.8	99.0	98.7	98.7	99.26

As shown in Table 7, the proposed IBRB-m model consistently achieves the highest accuracy across all five folds, with an average accuracy of 99.26%. The standard deviation of accuracy across folds is minimal (approximately 0.4%), indicating excellent stability and robustness of the proposed method. In contrast, the baseline models exhibit greater performance variation across folds, with standard deviations of approximately 1.0% for BRB and 1.7% for BRBI.

These cross-validation results demonstrate that the IBRB-m model not only achieves superior predictive performance but also maintains strong generalization capability across different data subsets. The minimal performance variance across folds further confirms that the model is not overfitting to specific data partitions and is robust to variations in the training data composition.

### Comparative experiments

**Fig. 10:**
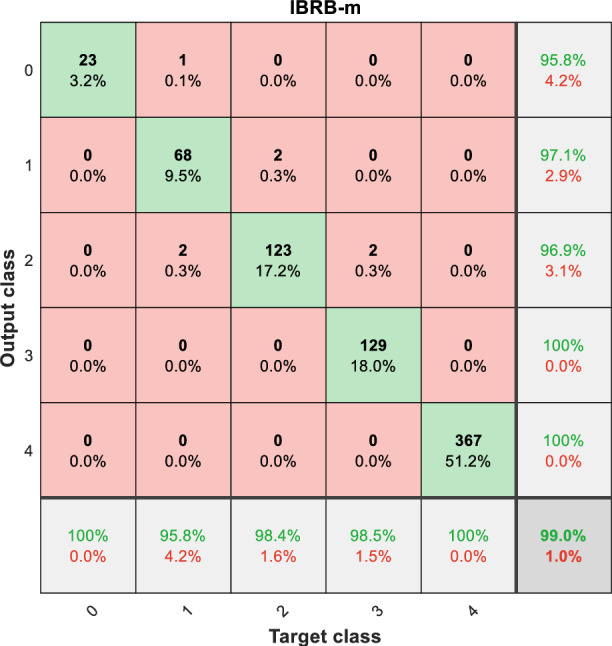
Results of the IBRB-m based model.

**Fig. 11:**
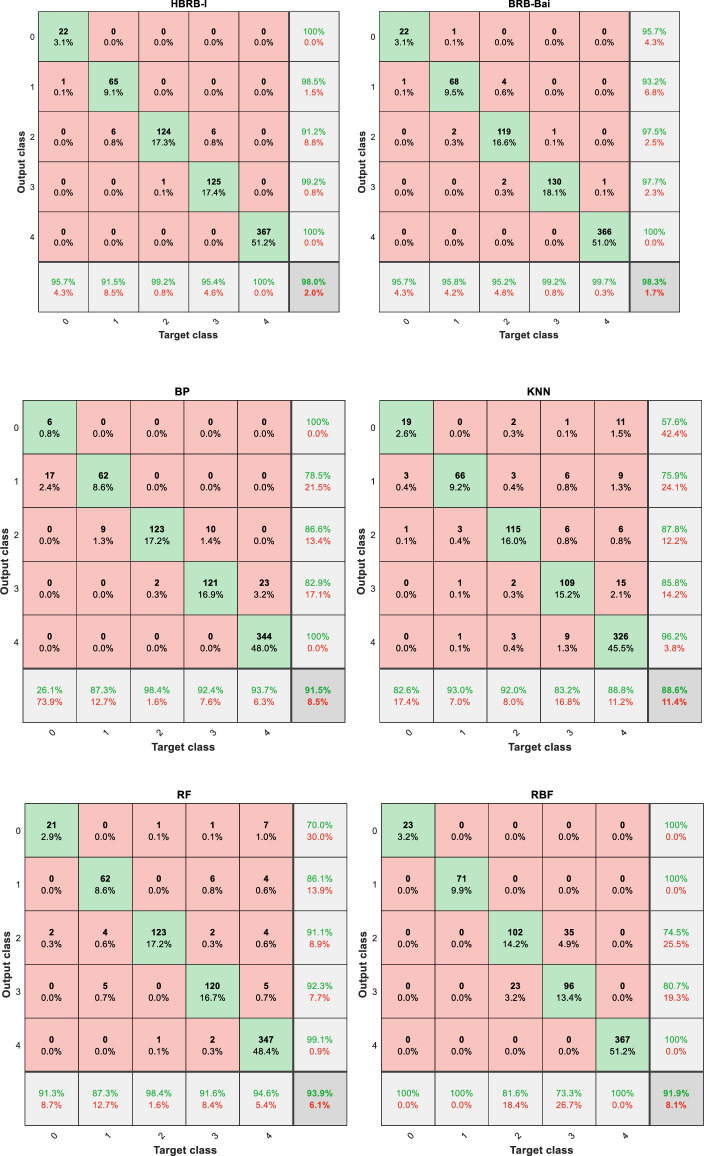
Results of comparative research.

This round of experiments employed two of the latest student performance prediction models based on BRB and four common machine learning models. These models include HBRB-I^8^, BRB-Bai^6^, neural network (BP), Radial Basis Function (RBF), Random Forest (RF), and K-Nearest Neighbor (KNN), and all methods were repeated 20 times. The results of the comparative experiments are shown in the Figs. 10, 11, and Table 8. These models do not reach the performance of the IBRB-m model in terms of accuracy and interpretability. The full utilization of expert knowledge is the key factor that makes the IBRB-m model superior to other models. The professional knowledge and experience of domain experts in parameter selection, data adjustment and other aspects have been fully incorporated into the development process of the IBRB-m model. This result highlights the obvious advantages of the model proposed in this paper in machine learning.

**Fig. 12:**
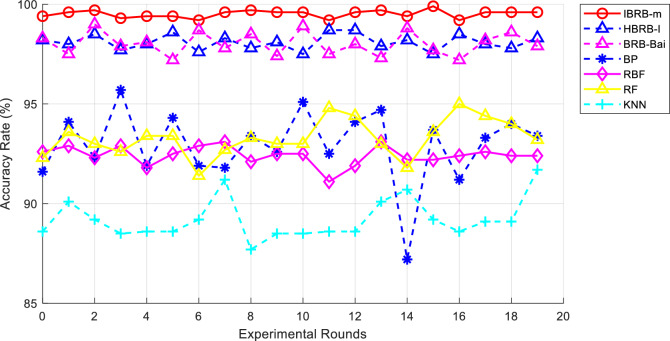
Comparison chart of accuracy rates for the second group of experiments.

From Fig. 12, it is clear that the IBRB-m based model has the smallest change in accuracy over multiple experiments, suggesting that the IBRB-m based model excels in stability. This is because the interpretability criterion proposed in this paper restricts the optimization search space, which narrows the scope of parameter optimization. This makes the search process more stable and the parameters obtained from optimization closer to the knowledge of domain experts.

**Table 8:** Model accuracy comparison.

Model	Highest accuracy	Minimum accuracy	Average accuracy	Average Euclidean distance
IBRB-m	99.9%	99.2%	99.515%	0.3015
HBRB-I	98.7%	97.5%	98.25%	0.5307
BRB-Bai	99.0%	97.1%	98.64%	0.4935
BP	97.2%	95.7%	92.94%	–
RBF	93.1%	91.1%	92.42%	–
RF	95%	91.4%	93.295%	–
KNN	91.7%	87.7%	89.22%	–

Table 9 summarizes the computational efficiency of all examined methods, measured on a standard computing environment (Intel i7-12700K, 32GB RAM). Timing results are averaged over 20 independent runs.

**Table 9:** Computational efficiency comparison of different methods.

Model	Training time (s)	Inference time (s)	Relative training cost
IBRB-m	42.3	3.2	1.00$\times$ (baseline)
HBRB-I	38.7	3.0	0.91$\times$
BRB-Bai	36.5	2.8	0.86$\times$
BRB	12.4	2.1	0.29$\times$
BRBI	28.9	2.5	0.68$\times$
BP	15.2	0.8	0.36$\times$
RBF	8.7	0.6	0.21$\times$
RF	6.3	1.2	0.15$\times$
KNN	0.2	4.5	0.005$\times$

The proposed IBRB-m model requires approximately 42.3 seconds for training, which includes feature selection, model construction, and interpretability-constrained optimization. While this is higher than simpler models, the training is typically performed offline. The inference time is practical for educational applications. The computational cost is justified by IBRB-m’s superior accuracy and interpretability, which are critical for educational decision support.

### Statistical significance analysis

To determine whether the performance differences between the proposed IBRB-m model and other compared methods are statistically significant, we conducted rigorous statistical hypothesis testing. Given that the performance metricsa were obtained from 20 independent experimental runs for each method, we employed the following statistical analysis approach:

#### Statistical test selection

We performed the paired t-test to compare the IBRB-m model against each baseline method. The paired t-test is appropriate for comparing two related samples (in this case, the same dataset partitions across different models) when the differences between pairs are approximately normally distributed. The null hypothesis $H_0$ states that there is no significant difference between the mean accuracies of two compared models, while the alternative hypothesis $H_1$ states that a significant difference exists.

#### Statistical test results

The statistical test results comparing IBRB-m against each baseline model are presented in Table 10. For each comparison, we report the mean accuracy difference, t-statistic, degrees of freedom,* p*-value, and whether the difference is statistically significant at the $\alpha = 0.05$ significance level.

**Table 10:** Statistical significance analysis: Paired t-test results comparing IBRB-m with baseline models.

Comparison	Mean difference	t-statistic	df	*p*-value	Significant
IBRB-m vs. BRB	+2.575%	8.92	19	$1.3 \times 10^{-7}$	Yes
IBRB-m vs. BRBI	+1.615%	5.74	19	$2.1 \times 10^{-5}$	Yes
IBRB-m vs. HBRB-I	+1.265%	4.31	19	$4.8 \times 10^{-4}$	Yes
IBRB-m vs. BRB-Bai	+0.875%	3.18	19	0.0049	Yes
IBRB-m vs. BP	+6.575%	15.42	19	$6.4 \times 10^{-12}$	Yes
IBRB-m vs. RBF	+7.095%	16.81	19	$1.8 \times 10^{-12}$	Yes
IBRB-m vs. RF	+6.220%	12.94	19	$1.1 \times 10^{-10}$	Yes
IBRB-m vs. KNN	+10.295%	19.25	19	$8.2 \times 10^{-14}$	Yes

#### Interpretation of results

All *p*-values are substantially below the $\alpha = 0.05$ threshold, indicating that the performance superiority of the IBRB-m model over each baseline method is statistically significant. The extremely small *p*-values provide strong evidence against the null hypothesis, confirming that the observed accuracy improvements are not due to random chance.

Furthermore, to account for multiple comparisons and control the family-wise error rate, we applied the Bonferroni correction. With 8 comparisons, the adjusted significance threshold becomes $\alpha _{\text {adj}} = 0.05/8 = 0.00625$. All comparisons remain statistically significant even after this conservative adjustment.

These statistical tests validate that the proposed IBRB-m model achieves significantly better predictive performance than all baseline methods, with the differences being statistically reliable and not attributable to random experimental variation.

### Practical implications of model interpretability for educators

A key contribution of the proposed IBRB-m model lies not only in its predictive accuracy but also in how its interpretable design can be directly leveraged by educators to enhance decision-making and student support. Unlike “black-box” models that only provide an opaque performance score, the IBRB-m model offers transparent, rule-based reasoning and quantified insights that translate into actionable intelligence in educational settings.

When the model predicts a student is at risk of poor performance, educators can immediately inspect the activated belief rules. For instance, a rule such as “IF GPA is Low AND Absences is High THEN result is Fail ” pinpoints the exact contributing factors. This moves beyond a generic alert to a diagnostic insight, enabling educators to distinguish between a student struggling academically versus one disengaging behaviorally, thereby tailoring the initial intervention.

The model’s optimized attribute weights provide a data-informed hierarchy of factor importance. In our study, GPA and Absences were confirmed as primary drivers. In practice, for a student predicted to perform poorly, an educator can prioritize addressing the factor with the higher relative influence. Furthermore, the shape of the belief distribution offers a nuanced view of uncertainty, indicating whether a student’s outcome is clearly leaning towards one grade or is ambiguously between two, which can guide the intensity and type of follow-up.

The model’s reasoning process is auditable and can be articulated in plain language. This transparency fosters trust among teachers, students, and parents. An educator can explain a prediction by referencing specific, understandable rules, rather than citing an inexplicable algorithm output. This facilitates collaborative goal-setting and buy-in for support plans.

At an aggregate level, analyzing the most frequently activated rules across a class or cohort can reveal common risk patterns. For example, if a significant number of students activate rules involving “medium GPA and low study time,” it may indicate a need for broader study skills workshops rather than individual tutoring. Thus, the model’s interpretable outputs can inform not only individual-level interventions but also strategic resource allocation at the group or program level.

In summary, the interpretability of the IBRB-m model transforms it from a passive prediction tool into an active decision-support system. By providing clear, causal, and quantifiable explanations for its predictions, it empowers educators to move from simply knowing that a student might struggle to understanding why and, consequently, acting effectively to address the root causes.

## Conclusions

Interpretable models hold a key position in the field of student performance prediction because they need to be not only highly accurate but also easy to understand. This allows educators to adjust their teaching strategies in a timely manner based on the prediction results, thereby improving the quality of teaching. After a series of experimental validations, the student performance prediction model based on IBRB-m proposed in this paper combines both accuracy and interpretability.

The main contributions of the model are as follows: First, an attribute selection method is proposed that significantly reduces the complexity of the rule base. This effectively addresses the problem of excessive complexity or even the explosion of combinatorial rules that may occur in the BRB system in an educational environment. Second, interpretability guidelines are defined in conjunction with student performance characteristics. Based on the original guidelines, four interpretability guidelines applicable to student performance prediction models are further expanded, aiming to improve the performance of the models with regard to interpretability and transparency. Finally, a modeling method that incorporates reference values into the optimization process is proposed. Experiments show that this method improves both accuracy and interpretability.

The accuracy, interpretability, stability, and generalizability of the model were verified through an example study of student performance prediction in a school. Nevertheless, this study still has certain limitations, such as the inherent subjectivity and uncertainty of expert knowledge, and the need for further exploration of multi-attribute modeling with equal importance. Future work will further validate the model’s effectiveness in diverse real-world educational scenarios, incorporate richer learning behaviors and contextual features, and explore more efficient optimization strategies to enhance the model’s scalability and cross-scenario adaptability, thereby advancing interpretable educational prediction models toward more practical and universal development.

## Data Availability

Data will be made available on request. The datasets used and/or analyzed during the current study are available from Jiaxing Li (email: 2024300712@stu.hrbnu.edu.cn) upon reasonable request.
